# Silicon and soil microorganisms improve rhizospheric soil health with bacterial community, plant growth, performance and yield

**DOI:** 10.1080/15592324.2022.2104004

**Published:** 2022-08-09

**Authors:** Krishan K. Verma, Xiu-Peng Song, Dong-Mei Li, Munna Singh, Jian-Ming Wu, Rajesh Kumar Singh, Anjney Sharma, Bao-Qing Zhang, Yang-Rui Li

**Affiliations:** a Key Laboratory of Sugarcane Biotechnology and Genetic Improvement (Guangxi), Ministry of Agriculture and Rural Affairs/Guangxi Key Laboratory of Sugarcane Genetic Improvement/Sugarcane Research Institute, Guangxi Academy of Agricultural Sciences/ Sugarcane Research Center, Chinese Academy of Agricultural Sciences, Nanning, China; b Department of Botany, University of Lucknow, Lucknow, India

**Keywords:** Plant microbes, environmental pressure, plant growth-development, productivity, stress resistance, sustainable agriculture, silicon

## Abstract

The interaction of silicon and soil microorganisms stimulates crop enhancement to ensure sustainable agriculture. Silicon may potentially increase nutrient availability in rhizosphere with improved plants’ growth, development as it does not produce phytotoxicity. The rhizospheric microbiome accommodates a variety of microbial species that live in a small area of soil directly associated with the hidden half plants’ system. Plant growth-promoting rhizobacteria (PGPR) play a major role in plant development in response to adverse climatic conditions. PGPRs may enhance the growth, quality, productivity in variety of crops, and mitigate abiotic stresses by reprogramming stress-induced physiological variations in plants via different mechanisms, such as synthesis of indole-3-acetic acid, 1-aminocyclopropane-1-carboxylate deaminase, exopolysaccharides, volatile organic compounds, atmospheric nitrogen fixation, and phosphate solubilization. Our article eye upon interactions of silicon and plant microbes which seems to be an opportunity for sustainable agriculture for series of crops and cropping systems in years to come, essential to safeguard the food security for masses.

## Introduction

Problems and concerns regarding the usage of synthetic fertilizers, irrigation, herbicides, and pesticides have prompted a quest for alternate techniques to combat nutrient and water limitations on crop plants. Microorganisms may boost crop nutrition as well as the ability of crops to withstand unfavorable environmental issues.^1–3^ Consequently, microorganisms in agricultural systems may reduce the application of traditional fertilizers, herbicides, and pesticides. The interactive demonstrations include bacterial and mycorrhizal stabilization of soil aggregates and morphology, rhizobacterial promotion of plant performance through enhancing the bioavailability of nutrients, and enhancement of plant development by influencing phytohormone levels and production of stress-defense components.^1,4,5^ Dodd et al.^6^ (2010) demonstrated changes in root hormones, morphology, and plant development mediated by rhizobacteria. Plant growth-promoting rhizobacteria (PGPR) mediated root trait variations may contribute to the agro-ecosystem via improving crop stand, resource use efficiency, stress resistance capacity with healthy soil profile.^7,8^

The long-term use of chemical fertilizers may adversely affect environment, soil health, quality, and production capacity of plants.^9^ The utilization of rhizospheric microorganisms was found to be beneficial for reducing the detrimental effects caused by synthetic fertilizers.^10,11^ Hence, useful plant–bacterial communities have been investigated for precision and sustainable agriculture. Diversified bacteria are closely connected with plant cells, and as a result, they extend beneficial effects.^12–14^ *Acetobacter, Acinetobacter, Azospirillum, Bacillus, Burkholderia, Candida boidinii*, Enterobacter, *Klebsiella variicola F2, Nocardiopsis alba, Penicillium chrysogenum, Pseudomonas, Promicromonospora, Pseudomonas fluorescens YX2, Raoultella planticola YL2*, and few others may colonize with broad range of plant cultivars.^14–20^ The distribution of endophytic bacteria is determined by various crops and crop circumstances.^21,22^ The endophytic bacteria on plants have a lot of potential as bio-fertilizers.^23^ Endophytic bacteria may get colonized intra and intercellularly promoting plant establishment and plant performance.^3,24,25^

The phyllosphere, endosphere, and rhizosphere are the different kinds of microorganisms.^26,27^ The phyllosphere, the above-ground surface of plants considered as complex ecosystem where microorganisms and the host plant get associated to create dynamic communities.^28^ The number of microbial species found in the microbiome of a live plant generally outnumbers the host cells.^14^ Plant microbiomes are important bioresources for sustainable agriculture as effective microbes may maintain plant development and enhance plant nutritional availability via solubilization of P, K, and Zn, fixation of nitrogen along with other functions.^29,30^ Agricultural practices, such as synthetic fertilizers and pesticides may change the composition of the plant microbiomes.^3^ Plants and microbes may also work together to reduce environmental pollutants and soil toxicity.^4^

Silicon is also considered somewhere between an essential and nonessential element for plants lifecycle, as it is not found essential for many crops. Still, plants get benefit for better adaptation toward adverse environmental conditions, in case having Si.^1,31,32^ Silicon constitutes a significant part of the soil, mainly aluminum silicates or other silicate minerals unavailable for plant absorption.^33^ The level of plant-available form of Si in the soil solution (H_4_SiO_4_) differs between 0.1 and .06 mM (2.8–17.1 mg Si L^−1^) is about two orders of magnitude higher than the phosphorus level.^34^ Silicon is taken up by plant root systems and translocated through the plant to be deposited as SiO_2_ phytoliths in the lumen, cell walls, and intercellular spaces.^35^ However, plant species vary greatly in their efficiency in accumulating Si ranging from 0.1 to 10% of Si on dry weight basis.^36–38^ Consequently, few plant species are less affected by Si application than others.^39^ Silicon regulates availability of various elements in soils by competing for binding on soil particles depending on the speciation of silicic acid.^40^ Soils contain 100–500 µmol L^−1^ silicic acid, although the availability varies depending on soil type, temperature, and pH level.^41,42^ Hence, it can be re-considered as a recently established definition of the importance of nutrients, according to Epstein and Bloom^43^ (2005), mostly found to be beneficial when plants face biotic and/or abiotic stresses.^31,44^

Our paper reports application Si and PGPRs essential to alleviating biotic and abiotic stresses and enhancing nutrient use efficiency (NUE) to promote plant performance and productivity. The recent advancement in Si and PGPRs interaction relies on the current scientific research to elucidate the benefits conferred by these bio stimulants in response to adverse environmental conditions for sustainable agriculture under climate change.

## Impact of Si and plant microbiome on plants

Implementing sustainable approach to increase plant resistance against environmental stresses is essential to enhance food production around the globe. The crop production may require ca. 60–100% enhancement by 2050 to meet the projected global population of 9.7 billion.^20,45^ However, achieving this target without any loss of agro-ecosystems is challenging. Nowadays, agricultural and food production sectors are threatened by the era of climate change, land degradation, limited availability of water, and more recently breakthrough in pandemics like COVID-19.^20^

## Biotic factors

Plants are always found in association with various species in natural habitats. They do have an inherent immune system that can detect invading microorganisms and create a possible successful response against specific phytopathogens. The plants’ innate immune system plays key role in regulating the action of microbial species in their microbiome system.^46,47^ Salicylic acid (SA) is produced in significant amounts by certain PGPRs, and some of these rhizobacteria may induce systemic resistance against stressed plants. Various PGPRs can produce SA in an iron availability-dependent way, and SA is detected on plant roots,^48^ although likely initiating from plant root systems upon interaction with rhizobacteria. Several PGPR strains have been reported to have the ability to produce SA. *Pseudomonas* is the best-studied genus for SA production, and SA-producing species include *P. aeruginosa, P. aureofaciens, P. corrugata*, and *P. fluorescens*.^49,50^ It has also been suggested that exudation of the signal molecules jasmonic acid (JA) and salicylic acid (SA) into the rhizosphere can be associated with the interplay between roots and microbes during the initial stage of colonization.^51–53^ Ethylene plays a vital role in stress tolerance capacity for some PGPR.^54^ As a result, plant microbiomes depend on the plant genotypes, growth age, and presence of bacterial strains.^53^

Bacteria are the most thoroughly characterized part of the plant microbiome due to their potential for plant growth promotion.^3^ Plant species, genotypes, life cycle duration, root proximity, and soil types influence the bacterial microbiome composition.^3,55,56^ Bacteria, the core of the complex interconnected microbiome, have a considerable impact on other microbiome residents, including other bacteria. The synergistic effects, such as biofilm development and bacteria cling with the surface protection.^57^ Furthermore, due to additive effects, multispecies biofilms are most efficient compared to monospecies biofilms, resulting in mutual proliferation when a new species is introduced to the microbiome.^58^ Olive trees cultivated with knot disease are an example of a significant association between bacteria in the plant microbiome. The pathogen was responsible for the olive knot found to be *Pseudomonas savastanoi PV. savastanoi* supplemented by nonpathogenic bacterial species like *Pantoea agglomerans* and *Erwinia toletana*, leading the disease to worsen.^59,60^ Bacterial interactions with other bacteria in the plant microbiome may be inhibiting. Many *Pseudomonas* species have also been reported to inhibit *R. solanacearum* and decrease pathogen density and infection prevalence.^61^ The intricate interplay in the plant, bacteria, and other microbes is more likely to impact the fundamental action mechanisms producing variations in the bacterial population of plants by other bacteria.^3,62^

Bacteria and fungi have been observed to interact extensively, with bacterial populations influencing fungal populations and vice versa. This relationship appears mostly plant-independent, as bacterial-mediated fungal community alterations have been observed in planted and unplanted settings.^63^ Depending on the precise condition of the interactions, bacteria in the plant microbiome might be advantageous or inhibiting to fungi. Through symbiosis, keystone bacteria, such as members of the *Burkholderia* genus, may considerably enhance the abundance of arbuscular mycorrhizal fungus (AMF), but *P. fluorescens* restrict fungal growth functions.^64^ In-plant microbiome, symbiosis is commonly associated with bacteria and fungi, notably with AMF.^4,64,65^ *Rhizopus* spp. of fungi and bacterial spp. *Burkholderia* developed an endosymbiotic relationship in which *Rhizopus* fungi relied on *Burkholderia* activities to create spores that infected Oryza plants with blight.^66^ Fungi play various important functions in the plant microbiome. The plant microbiome’s fungi vary by host, and get influenced by nitrogen deposition,^67^ and soil types.^68^ Fungi may be significant to plant development and cause plant diseases,^69^ also enhances plant performance, including productivity.^70^

*Archaea* have more potential for plant growth enhancement, despite being understudied concerning the plant microbiome. Their close connections with atmospheric variables and other microbial residents frequently form symbiotic relationships with plants.^1,71^ *Archaea* were revealed to interact with plants and fungus in a bog habitat, impacting nutrient delivery and secondary plant capabilities. *Archaea* have not been thoroughly explored with their possible application in agriculture. The archaeon *Methanococcoides burtonii* produces non-photosynthetic Rubisco (Ribulose-1,5-biphosphate carboxylase), rate-limiting CO_2_ fixation process during leaf gas exchange, which was found to boost photosynthetic performance and plant development capacity in tobacco plants by acquiring in vivo biosynthesis of rubisco encoded by *rbc*L and *rbc*S genes of cpDNA and nDNA, respectively.^72^ This response suggests a link between nematodes and the various players in the plant microbiome, which might be used in agriculture to protect against nematode infection.^73^

Silicon and PGPRs have been shown to mitigate adverse plant diseases.^1,74^ Some of the known mechanisms by which Si and PGPRs alleviate the biotic stress in plants are shown in Table 1 and Table 2. Many suitable nutrient-rich niches on/inside roots attract a greater diversity of microorganisms. The basic process by which PGPRs defend plants from phytopathogens is competition for the nutrients and niches.^39,143,144^ Induced systemic resistance, which improves defense against a wide range of diseases and insect herbivores, is another meaningful way pathogens control PGPRs in the rhizosphere and endorhiza prime the entire plant body.^1,4,143,145^ Like PGPRs, Si may manage plant pathogenic diseases by forming physical barriers. Precipitation of amorphous silica in plants is a mechanical barrier.^146^

**Table 1.** Impact of silicon on plant biology subjected to stress conditions.

Stress condition	Crop	Impacts	Source
Water-deficit	*Saccharum officinarum*	Increased photosynthetic capacity, pigments, leaf water status, phytohormones, and antioxidative enzyme activities	^1,75–78^
	Strawberry (*Fragaria x ananassa ‘Camarosa*’)	Improved leaf development, injury of membranes, SPAD units, chlorophyll fluorescence variables, leaf gas exchange and biomass traits	^79^
	*Solanum lycopersicum*	Photosynthetic performance, membrane injury, enzymatic and non-enzymatic activities and growth parameters increased	^80^
	*Triticum aestivum, Zea mays*	Enhanced plant biomass and photosynthetic and productivity	^81,82^
Saline stress	*Cucumis sativus*	Plant biomass, enzymatic and non-enzymatic activities increased with plant development	^83^
	*Oryza sativa*	Growth-biomass, photosynthetic CO_2_ assimilation rate, ion distribution improved or balanced	^84^
	*Vigna radiata*	Plant productivity, pigments, photosynthesis and stress resistance capacity enhanced	^85^
Osmotic	*Sorghum bicolor*	Leaf photosynthesis responses, root hydraulic conductivity and biomass capacity improved	^86^
	*Solanum lycopersicum*	Gas exchange rate, leaf water status, MDA, hydrogen peroxide content and enzymatic responses increased during stress with Si application	^80^
Excess Cd	*Oryza sativa*	Increased plant weight and enzymatic activities in leaf and roots	^87^
Excess As	*Oryza sativa*	Improved leaf photosynthetic, fluorescence variables and Vcmax and Jmax responses	^88^
Excess Cu	*Spartina densiflora*	Photosynthesis, pigments, relative growth rate and biomass upregulated	^89^
Excess Mn	*Cucumis sativa*	Biomass, leaf mineral content, H_2_O_2_ and GPx level enhanced	^90,91^
Excess Al	*Zea mays*	Root development, citrate and malate exudation and phenol exudation increased or upgraded	^92^
Low K^+^	*Sorghum bicolor*	Increased whole plant biomass, photosynthesis assimilation rate of leaf, chlorophyll level and enzymatic activities	^93^

**Table 2.** Impact of soil microbes on agricultural crops subjected to abiotic stressors.

Stress	Plant	Microbe	Function	Source
Excess temperature	*Solanum tuberosum*	*Paraburkholderia phytofirmans*	Production of ACC deaminase	^94^
	*Solanum lycopersicum*	*Mycorrhizae*	Decrease MDA and H_2_O_2_ content, enhance ROS activity in plant organs	^95^
	*Triticum aestivum*	*Bacillus amyloliquefaciens*, *Azospirillum brasilense*	Reduce ROS, pre-activation of heat shock proteins (HSPs)	^96^
	*Glycine max*	*Bacillus aryabhatthai* SRBO2	Production of ABA content	^97^
	*Triticum aestivum, Sorghum bicolor*	*Pseudomonas putida* AKMP7, *Pseudomonas* sp. AKMP6	ROS reduce, enhance proline, photosynthetic pigments, sugar, starch, amino acid and protein and plant hormones	^98^
Low temperature	*Malus pumila, Pyrus communis*	*Ps. fluorescens* A506	Interaction with INA+ bacteria	^99^
	*Triticum aestivum*	*Pantoea dispersa* 1A, *Serratia* *marcescens* SRM, *Pseudomonas* spp. PGERs17, NARs9	Production of ACC deaminase	^70,100–102^
	*Vitis vinifera*	*Paraburkholderia phytofirmans*	Production of ACC deaminase	^103,104^
Drought	*Cucumis sativus, Phaseolus vulgaris*	*Phoma glomerata, Penicillium* sp., *Exophiala* sp., *Paecilomyces formosus*, *Glomus intraradices*	Improve soil properties and root water conductivity	^105,106^
	*Solanum lycopersicum, Cucumis sativus, Citrus x sinensis*	*Pseudomonas chlororaphis* TSAU13, *Funneliformis mosseae*	Production of IAA	^107,108^
	*Cucumis sativus*	*Burkholderia, Promicromonospora*, *Acinetobacter, Pseudomonas* spp.	Production of gibberellin	^109^
	*Lactuca sativa*	*Bacillus subtilis*	Production of cytokinin	^110^
	*Glycine max*	*Ps. putida* H-2-3	Production of ABA	^111^
	*Solanum lycopersicum, Piper nigrum, Pisum sativum, Arachis hypogaea*	*Achromobacter piechaudii* ARV8, *B. licheniformis* K11, *Pseudomonas* spp., *Ps*. *fluorescens* TDK	Production of ACC deaminase	^112–115^
	*Daucus carota, Glycine max, Lactuca sativa, Ocimum basilicum*	*Glomus intraradices*	Increase aquaporin activities	^116,117^
	*Ocimum basilicum*	*Pseudomonas* sp.	Up-regulated antioxidant enzyme activities	^118^
	*Helianthus annuus, Citrus x sinensis*	*Ps. putida, Ps. aeruginosa* PF23, *Glomus* *mosseae, G. versiforme*, *G. diaphanum*	Production of EPS	^119–121^
	*Solanum lycopersicum, Glycine max*, *Cirus reticulata*	*Bacillus polymyxa, Glomus intraradices*, *G. versiforme*	Production of osmolytes	^119,122–124^
Salinity	*Zea mays*	*Azospirillum*	Osmoprotection	^125^
	*Lactuca* *sativa*	*Azospirillum*	Increase germination rate	^126^
	*Brassica* *napus*	*Pseudomonas putida* UW 4	Upgrade plant growth, development and yield biomass	^127^
	*Arachis* *hypogaea*	*Pseudomonas* *fluorescens*	Increased ACC deaminase activity	^113^
	*Zea mays*	*Pseudomonas syringae*, *P. fluorescens*, *Enterobacter aerogenes*	Enhanced ACC deaminase level	^128^
	*Phaseolus* *vulgaris*	*Azospirillum brasilense*	Improve root morphological capacity and increase secretion of nod-genes	^129^
	*Zea mays*	*Rhizobium*, *Pseudomonas*	Decrease EC content and increase proline level, balance RWC status in leaves, and accumulation of K ions	^130^
	*Triticum aestivum*	*Pseudomonas sp.*, *Serratia sp*	Enhance ACC deaminase activity	^131^
	*Gossypium* sp.	*P. putida Rs-198*	Enhance the absorption of Mg2+, K2+, and Ca2+ and reduce Na2+ uptake from soil	^132^
	*Vigna* *radiata*	*P. syringae*, *P. fluorescens, and* *Rhizobium phaseoli*	Increase ACC deaminase content	^133^
Metal toxicity	*Pisum sativum*	*Enterobacter* sp.	Restored growth injuries by reducing ion distribution. Enhanced growth performances and chlorophyll level	^134^
	*Lens culinaris*	*Bacillus* sp.	Minimize the harmful effects of Cr on plants and enhanced plant performance and development	^135^
	*Vinca rosea*	*Bacillus megaterium* MCR−8	Increased growth characteristics, proline and total soluble protein level during metal toxicity applied media and upregulated antioxidative enzymatic activities	^136^
	*Brassica nigra*	*Kocuria* sp. CRB15	Enhanced plant growth and development and reduced contaminated ion uptake and accumulation in plant organs	^137^
	*Oryza sativa*	*Klebsiella pneumoniae* MCC 3091	Enhanced seedling rate of germination and biomass capacity during stress. Upgraded the level of photosynthetic pigments, enzymatic and non-enzymatic activities and downregulated the production of ethylene during metal stress condition.	^138^
	*Panicum virgatum*	*Azospirillum*	Enhanced the growth parameters, biomass and balance the pH of soil and protect the accumulation of toxic ions uptake	^139^
	*Solanum nigrum*	*Pseudomonas* sp. LK9	Upgraded the morphological traits with yield productivity. Positively enhanced or balanced the uptake of Cu, Zn, and Cd ions in plant organs. Increased the level of P and Fe in soil properties.	^140^
	*Sedum plumbizincicola*	*Achromobacter* sp. E4L5, *Bacillus* sp. E4S1, *Bacillus* sp. E1S2, *Bacillus pumilus* E2S2, *Stenotrophomonas* sp. E1L	Significantly enhanced the morphological and biomass activities of plants during stress condition.	^141^
	*Eruca sativa*	*Pseudomonas putida* (ATCC 39213)	Significantly enhanced the growth, biomsss and development of plants and leaf photosynthetic pigments. Cd ion uptake increased.	^142^

1-Aminocyclopropane-1-carboxylic Acid (ACC), Ice Nucleating Activity (INA^+^), Abscisic Acid (ABA), Reactive Oxygen Species (ROS), Indole-3-acetic Acid (IAA), Abscisic Acid (ABA), Exopolysaccharides (EPS)

Various chemical changes in soil are linked with PGPRs. Some bacterial strains directly associate plant physiology by mimicking the synthesis of phytohormones, whereas others enhance the availability of minerals and nitrogen content in the soil to augment growth.^147^ Another effect of Si on pathogenic disease control to the formation of chemical barriers, that is, the increased defense-related enzymes such as chitinases, β-1,3-glucanases, peroxidase, lipoxygenase, polyphenol oxidases, and phenylalanine ammonia-lyase.^1,2^ Silicon may enhance plant resistance capacity to pathogenic infections by (i) enhancing gene expression associated with tolerance to pests and diseases; (ii) increasing phenolic content, callose, or methylaconitate (phytoalexins), and lignin; (iii) upregulating the expression of genes associated in encoding the proline-rich protein (PRP1) and the major enzymes in phenylpropanoids pathways; and (iv) increase polyphenol content, antimicrobial flavonoids, and anthocyanin.^1,2,39,144,148^

## Role of plant rhizobacteria and Si on plants in response to adverse environmental conditions

### Water and salinity stress

Water stress is a major environmental stressor in agro-ecosystems, resulting in losses ca. millions of dollars annually.^1,75,149^ However, water stress on plants has been extensively researched, but less information about the effect of drought on the plant microbiome. Water stress, of course, causes a complete loss of plant and microbial biomass.^150,151^ However, investigations on diverse plants utilizing stress treatments that resulted in an enormous enrichment in bacteria have indicated that some species of bacteria do a better performance during stress conditions.^144,151^ *Acinetobacter* and *Pseudomonas* bacterial isolates alleviated plant biomass declines in water deficit-stricken grapevines by reducing photosynthetic inhibition caused by stress, most likely through 1-aminocyclopropane-1- carboxylate (ACC) deaminase production. The proposed mechanisms for plant-growth enhancement by PGPRs include bacterial synthesis of the phytohormones indole-3-acetic acid (IAA), cytokinin (CYT), and gibberellin (GA); breakdown of plant-produced ethylene by bacterial production of ACC deaminase; and enhanced the availability of nutrients and nitrogen in the soil.^3,147,152,153^

On the other hand, these bacterial strains did not enhance plant development in normal plants^153^ while in case infected with *Bacillus thuringiensis* IAM 12077 in subtropical climates then found that it improved plant performance and nutritional absorption.^154^ Generally, bacteria enhance plant water resistance by boosting root water intake, lowering ethylene production, and enhancing metabolism and nutrient synthesis.^3,4^

Salinity is a severe problem for crop production, and it has negatively affected plant health by generating nutrient absorption imbalances by changing osmotic pressures in plant cells.^155^ Due to the drying and lysis of microorganisms in the plant microbiome, salinity substantially impacts microbiome composition since halophytic bacteria tend to resist extremely saline soils.^1,156^ Salinity decreases mycorrhizal colonization, hyphae growth, and germination efficiency, inhibiting fungal growth. The plant microbiome is a complex network of interdependent bacteria that shifts many other factors interacting with fungus. Compared to fungi, the effect of salinity appears to have a more significant influence on bacteria.^157^ Because most bacteria are not acclimated to highly saline conditions, rising soil salinity drastically reduces bacterial richness and diversity.^157,158^ Plant growth-promoting bacteria have been identified from rhizospheric saline soil and low potential for increasing plant development in salt-stressed areas in an environmentally acceptable approach.^1^ Fatima et al.^159^ (2020) found that the saline-soil isolate *Alcaligenes* sp. AF7 had a variety of plant growth-promoting traits, such as the production of exopolysaccharides, indole-3-acetic acid, gibberellic acid, and siderophores that were prominent up to various salinity thresholds, and the bacterium enhanced the Oryza vegetative growth stage more than two-times during salinity.

The strain NCCP-11 T of *Cellulomonas pakistanensis* sp. nov., isolated from paddy shown to be moderately halotolerant.^160^ *Cellulomonas* isolates found potential as PGPR in rice, boosting minerals bioavailability by decomposing organic matter via secreted enzymes, that is, cellulases and hemicellulases.^161^ Most of the trials under salinity-stress conditions could find other important halotolerant microorganisms in the microbiome to be used during salinity to sustain agricultural crop production. Plants with Si and PGPRs are beneficial for the salinity and water-stressed conditions.^1,85,162^ Tables 1 and 2 highlights the action mechanisms through which Si and PGPRs help plants cope with saline and water-stressed plants. By limiting the accumulation and distribution of water and minerals, salinity and water deficit negatively affect plant development, development, and output.^163^ The length and region of the root surface affect plant nutrient absorption and more exposed areas for the absorption of scattered ions result from an increase in root surface morphology. During salinity and water stress conditions, Si can improve root development, nutrient uptake, phytohormones, and overall plant performance.^144,164,165^

The water deficiency also restricts nutrient uptake and accumulation by roots and subsequent transfer to shoots, lowering nutritional bioavailability and metabolism.^166^ PGPRs may directly affect plant behavior by solubilizing P by secreting a variety of extracellular phosphatases and producing organic and inorganic acids and protons and K to improve nutrient uptake.^1,74^ PGPRs may enhance plant resistance capacity to salt and water deficit by reducing Na^+^ absorption and affecting the accumulation and distribution of a few primary nutrients in plants treated with Si.^167^ In earlier demonstrations, the exogenous use of Si increased leaf photosynthetic performance in various plant cultivars during salt and limited water supply.^75^ Higher levels of soluble salts and specific ions (Na^+^ and Cl^−^) severely influence plant performance by enhancing osmotic pressures and inhibiting water absorption and distribution.^39,163^ Plants require an optimal water level for survival during salinity and water stress conditions via osmotic adjustment.^168^

The incredible action of Si is achieved by adjusting the levels of solutes, such as proline, glycine betaine, carbohydrates, and polyols and antioxidative enzymes, that is, total phenolics, total soluble sugars, and total-free amino acids caused by salinity. Reactive oxygen species play important roles in balancing normal plant development and improving their tolerance efficiency to stresses.^31,39,144^ Damage to the plasma membrane and endomembrane systems and disruption of normal metabolism can result from these ROS. Like PGPRs, Si-fertilizer may reduce oxidative injury in various plants during salinity and water stress by increasing antioxidative enzymatic and non-enzymatic activities.^169^ Further, Si maintained the permeability and stability of cell membranes in plants cultivated during abiotic stress.^39^ Reduced leaf relative water status is a common reaction of plants in salinity stress circumstances.^1,32,164,170^ Various scientific reports have also shown that the Si may significantly enhance plant tolerance capacity to noxious environmental factors.^31,32^ Inoculating stressed plants with PGPRs may activate signaling pathways that increase the host’s disease resistance, the phenomenon known as induced systemic resistance.^148,171^ PGPR-mediated physical and chemical variations that improve resistance to stressors have also been proposed as induced systemic tolerance.^8,172^ Silicon leads to activating key genes associated with salinity and drought-stressed plants.^39,173^

## Heavy metal toxicity

When agricultural lands are contaminated with heavy metals (HMs), soil microorganisms are more sensitive to the impacts of HMs.^174,175^ The number of soil microorganisms reduces by direct killing or biochemical deactivation. Heavy metal pollution will change the composition of the soil microbial community, and the microorganisms that can adapt to these stress increase in abundance.^175–177^ The different HMs have different effects on the microbiome, with zinc being the more effective in causing alterations in microbial diversity, followed by cadmium and lead.^178,179^ Although the impact of metals on different members of the microbiome are not fully understood, the application of plant growth-promoting microbes to aid in contaminated metallic ions reduced through phytoextraction has the potential to become broadly used in agriculture due to its demonstrated effectiveness.^180,181^ These strains could be employed to enhance plant performance in metallic-contaminated environments. The effects of toxic ions on the microbiota may identify new players who could help bioremediation efforts in agricultural systems.^8^

Metal–resistant siderophore–producing PGPRs might reduce metallic toxicity by providing nutrients to plants and binding metals other than Fe.^4^ Plant hormones, i.e., auxins, gibberellins, and cytokinin-producing-PGPRs, may mitigate metal induced-stress in plants and facilitate adaptation and resistance strategies by triggering physiological variations and boosting the absorption of important minerals as a result of expanding plant root systems.^8^ Interactive use of Si and PGPRs may reduce toxic effects on plants exposed to metal toxicity.^1^ A variety of plants were shown to have increased tolerance capacity to harmful toxic ions by reducing the accumulation and distribution of metals.^182,183^ Under various stresses, PGPRs stimulate HMs tolerance genes as well as the development of more stress response functions, such as ETH and stress proteins.^3,184,185^

## Nutrient deficiency

In plants, Si and PGPRs were found to minimize the negative impacts of nutrient deficiency.^1,186^ Tables 1 and 2 highlights some known processes by which Si and PGPRs restore nutritional imbalance in plants. Si also helps plants to cope with N deficiency by (i) increasing nutrient absorption, (ii) improving nodulation and N_2_ fixation in leguminous crops, (iii) increasing NUE, and (iv) changing primary metabolism by driving amino acid remobilization.^1,39,144^ Silicon fertilization was discovered to boost P availability in graminaceous species. At the same time, Si may inhibit P uptake and formation of chlorosis when excessive P levels are applied, presumably by minimizing the rate of transpiration.^144^ Si also reduces stress caused by K shortage by regulating soil K availability and nutrient content in plants and altering antioxidative enzyme activities to reduce K-deficiency-induced MDA content and oxidative stress.^187–189^

PGPRs enhance plant micronutrient availability,^14^ reducing soil pH and synthesizes chelating agents. Plants require iron as a key micronutrient element in dry, calcareous, and alkaline environments. Microbial synthesis of the plant hormone auxin, i.e., indole-3-acetic acid/indole acetic acid/IAA, has been known for long period. Generally, IAA secreted by rhizobacteria interferes with the various plant developmental processes because the endogenous pool of IAA can be altered by the acquisition of IAA that has been secreted by soil bacteria.^190,191^

IAA also acts as signaling molecule affecting gene expression in several microorganisms. It plays vital role in PGPRs-plant interactions. Most *Rhizobium* species have shown to produce IAA.^192,193^ Environmental stress factors that modulate the IAA biosynthesis in different bacteria include acidic pH, osmotic and matrix stress, and carbon limitation.^191^ Generally, bacteria acquire iron by the secretion of low-molecular-mass iron chelators referred as siderophores, which have high association with iron. PGPRs vary regarding the siderophore cross-utilizing efficiency; some are proficient in using siderophores of the same genus (homologous siderophores), while others could utilize those produced by other rhizobacteria of different genera (heterologous siderophores).^193,194^ Hence, bacterial siderophores may help to mitigate adverse environmental stresses. Plants assimilate iron from bacterial siderophores utilizing different mechanisms, for instance, chelation and release of iron, the direct uptake of siderophore-Fe complexes, or ligand exchange reaction.^195^ Numerous studies of the plant growth enhancement via siderophore-mediated Fe-uptake.^4,196,197^

### pH

The pH of the soil is a key component in defining the composition of plant microbiome and its impact on heavy metal accumulation. Bacterial species have shown significant fluctuations with little changes in pH, but fungus types found to be slightly associated.^198–200^ The highly alkaline soils impair plant growth due to poor mineral uptake while acidic soil may cause injury due to excessive uptake of toxic ions.^201,202^ Some microorganisms that promote plant growth have shown promise in reducing alkaline stress in plants. Dixit et al.^203^ (2020) isolated different PGPRs such as *Alcaligenes* sp. NBRI NB2.5, *Bacillus* sp. NBRI YE1.3, and *Bacillus* sp. NBRI YN4.4 from alkaline soil and discovered that they improved germination efficiency and productivity of maize plants *in vitro* condition during alkaline stress with *Bacillus* strain NBRI YN4.4 having most remarkable enhanced plant growth. The soil inoculation with NBRI NB2.5, NBRI YE1.3, and NBRI YN4.4 enhanced enzyme activities in the soil, such as *alkaline phosphatase, beta-glucosidase*, and *dehydrogenase* under alkaline conditions, indicating the importance of these strains for plant performance and soil fertility in alkaline conditions.^203^

## Role of plant hormones with the application of plant microbes and silicon

In addition, endophytic bacteria promote plant growth by producing several phytohormones. Plant nutrient uptake and biomass get boosted by phytohormones.^204,205^ Endophytic bacteria create plant growth regulators like CYT, ETH, ABA, GA, and IAA during plant interactions.^1,206^ Surprisingly, IAA has a significant impact on overall plant performance. Indole-3-acetic acid is one of the main signals in symbiotic relationship between host and endophytes.^207^ Ethylene is also an important plant hormone that regulates variety of developmental and physiological functions.^208^ Endophytic bacteria produce ethylene precursor enzyme 1-aminocyclopropane-1-carboxylate (ACC) deaminase.^180,209^ Under stressful conditions, ACC hydrolysis improves plant development^24^ while, some endophytic bacterial strains may emit volatile organic substances like acetoin, pentadecane, 2,3-butanediol, 1 hexanol, and indole, promoting plant development.^147,210,211^ Under water stress, phytohormone producing-PGPRs have also been shown to enhance plant resistance against various environmental stresses by changing phytohormone contents viz., auxins, ETH, GA, CYT, and ABA.^1,39,212,213^ The generation of IAA by PGPRs alters root morphology by enhancing the number of root tips and surface area, acquisition of water and minerals with an ability to cope up the drought.^32,212^

## Agricultural approaches

### Crop rotation and fertilizer use

Crop rotation, applying natural and synthetic fertilizers and using microbes or transgenic plants are some of the advanced agricultural practices utilized by farmers these days to boost the plant performance, productivity, and quality of the crops including disease-resistant soil^214^ with improved rhizospheric soil.^215^ However, the impact of crop rotation on microbial populations in the soil has been poorly known for a long time.^216,217^ Arbuscular mycorrhizal fungi (AMF) and PGPR have been used as biofertilizers for their beneficial effects on plant development, especially by mobilizing soil nutrients and producing plant hormones that stimulate root growth.^218^ Long-term demonstrations may be used to assess the sustainability of intensive cropping systems about the effect of continuous fertilization in different combinations of nutrients on the soil profile and crop yield.^219^ The concentration of amorphous Si sometimes ranged from less than 1–30 mg g^−1^ on a total soil basis. Crops may remove millions of tons of Si from soil annually around the world.^183,220,221^

Microbial activity and bio-mineralization play a major role in nutrient cycling in soil and may be shifted by long-duration fertilization.^222^ Cultivation of some crops may also be an alternate source of soil fertility.^223^ The downregulating behavior of plant-available silicon affected by crop rotations is accompanied by N, P, and K combined fertilization. The optimum mineral NPK application can increase the Si-use efficiency (SUE) and promote its uptake/accumulation by plants.^221^ However, the fertilization by Si can still be an important demand to replenish the reduced levels of nutrients depending on its critical levels in soil and the plant nutrient requirements.^224,225^ For many years, farmers have used natural and traditional fertilizers on their crops. These fertilizers’ made impact on the soil’s microbial composition, not well understood.^226^ On the other hand, high nitrogen fertilization enhanced abundance of nitrification and denitrification genes in the surrounding soil with an ability to improve crop biomass and nitrogen content. Lang et al.^227^ found that when phosphorus fertilizer increased, the overall community richness of AMF and bacteria dropped, while fungal and bacterial gene copies may get enhanced.

## Limitations and concluding remarks of the study

As a result of advancements in DNA sequencing methods, studying the microbiome has become an important research topic these days. The breakthrough may explore better understanding of complicated microbial communities.^228^ It may reveal wealth of knowledge about micro-ecosystems that occur inside soil by studying the community of bacteria, culturable and unculturable in natural surroundings.^229^ Targeted amplicon gene sequencing or metagenomic shotgun sequencing are commonly used to investigate these communities through DNA sequencing^230^ with constraints to the soil environment as it is low-cost and effective tool to acquire relevant data of microorganisms. The interplay between Si and the plant microbiome seems to be one the fascinating current studies, also expanding these days with pace. The internationally harmonized regulatory framework (ISO/TC-134) is working on the standardization in the field of fertilizers, soil conditioners, and beneficial substances, that is, materials whose addition is intended to ensure or improve the nourishment of cultivated plants and/ or to improve the properties of soils, and the efficient use thereof. Other key topics include exploration of beneficial mechanisms of Si and bacteria, their plant growth-promoting properties, and the best way to recruit or employ a healthy microbiome consortium to boost crop improvement, cropping systems and plant productivity for sustainable agriculture for fast growing population, globally.
